# Effect of calcium electroporation in combination with metformin *in vivo* and correlation between viability and intracellular ATP level after calcium electroporation *in vitro*

**DOI:** 10.1371/journal.pone.0181839

**Published:** 2017-07-25

**Authors:** Stine Krog Frandsen, Julie Gehl

**Affiliations:** Center for Experimental Drug and Gene Electrotransfer, Department of Oncology, Herlev and Gentofte Hospital, University of Copenhagen, Herlev, Denmark; Universidad de Navarra, SPAIN

## Abstract

**Background:**

Calcium electroporation is a new experimental anti-cancer treatment where calcium is internalized into cells by application of short, high voltage pulses. Calcium electroporation has been shown to induce tumor necrosis associated with ATP depletion while the effect on normal fibroblasts was limited when investigated in a 3D *in vitro* spheroid model. We aimed to investigate the effect of calcium electroporation in combination with metformin, a drug that affects intracellular ATP level. We also aimed to study the relationship between the viability and intracellular ATP levels after calcium electroporation *in vitro*.

**Methods:**

In this study, we investigated the effect of calcium electroporation with metformin on NMRI-*Foxn1*^*nu*^ mice *in vivo* on tumor size, survival, and intracellular ATP. We further investigated viability and intracellular ATP level *in vitro* after calcium electroporation in two human cancer cell lines: Breast (MDA-MB231) and colon (HT29), and in normal human fibroblasts (HDF-n), as well as investigating viability in human bladder cancer cells (SW780) and human small cell lung cancer cells (H69) where we have previously published intracellular ATP levels.

**Results:**

Calcium electroporation significantly reduced the size and ATP level of bladder cancer tumors treated *in vivo* but no increased effect of metformin combined with calcium electroporation was shown on neither tumor size, survival, nor ATP level. Calcium electroporation *in vitro* significantly decreased viability compared with calcium alone (p<0.0001 for calcium concentrations from 0.5 mM for H69, HDF-n, and MDA-MB231; p<0.0001 for calcium concentrations from 1 mM for HT29 and SW780). Intracellular ATP levels decreased significantly after calcium electroporation (p<0.05), however no correlation between intracellular ATP level and viability after treatment was observed.

**Conclusion:**

Calcium electroporation caused reduced tumor size, increased survival, and acute ATP depletion *in vivo*. This effect was not augmented by metformin. Calcium electroporation is a possible novel anti-cancer treatment that has been shown to cause cell death associated with acute ATP depletion *in vitro* and *in vivo*.

## Introduction

Electroporation is a method where application of short, high voltage pulses causes transient permeabilization of the cell membrane, letting ions and molecules enter and leave the cell [1–3]. Electroporation based treatments include electrochemotherapy, where chemotherapeutic agents are internalized into tumor cells, because permeabilization of the membrane by electroporation allows direct access to the cell cytosol [4–9]. Electrochemotherapy is in clinical use for cutaneous tumors [10–16], and in development or clinical trials for tumors situated in e.g. bone [17], pancreas [18], brain [17], the colon/rectum [19], as well as head- and neck cancers [20]. Irreversible electroporation [21–23] is also used clinically, and with this technology permeabilization of the cell membrane is much more pronounced, so that tumor cells are extensively permeabilized, leading to cell death associated with loss of ionic homeostasis, and energy depletion. Finally, nanosecond pulses have been shown to induce cell death [24, 25] by permeabilizing internal organelles such as the mitochondria, and this technology is also entering clinical trials.

A novel use of electroporation is calcium electroporation, where high calcium concentrations are introduced into the cell cytosol, and this has been proposed as a potential novel anti-cancer treatment [26]. Calcium is an important ubiquitous second messenger involved in numerous cellular processes [27–30], and can be involved in development of the malignant phenotype [31, 32]. Calcium electroporation has been studied *in vitro* [26, 33, 34], in 3D spheroids [35], and has previously been shown to induce tumor necrosis *in vivo* [26], and a clinical trial treating cutaneous metastases is ongoing (ClinicalTrials.gov ID- NCT01941901). Calcium electroporation has also been shown to be associated with acute ATP depletion [26, 35, 36]. Thus, it could be assumed that combination of calcium electroporation with another treatment affecting the cell metabolism and thereby the intracellular ATP level might have an additive effect. Such a treatment could be metformin, a biguanide that reduces blood glucose, and is a commonly used drug for treatment of type 2 diabetes. Metformin exerts a number of effects including reducing the ATP production by binding to and inhibiting complex I of the mitochondrial respiratory chain [37]. Thus, the mitochondrial ATP production is inhibited by metformin causing a reduced intracellular ATP level, leading to changed ATP/AMP ratio which activates AMP-activated protein kinase (AMPK). This activation causes inhibition of cell survival, growth, and proliferation; thus, contributing to an anti-cancer effect [37–40].

We hypothesized that the ATP reducing effect of metformin and calcium electroporation could be additive. The aim of this study was therefore to test the effect on viability, tumor size, and ATP levels in tumors, after treatment with systemic metformin in combination with local treatment with calcium electroporation on a nude mouse model. We also aimed to determine the relationship between viability and ATP depletion after calcium electroporation, in a number of different cell lines *in vitro* in order to investigate possible differences in sensitivity and effect.

## Methods

### Cell culture

Four different human cancer cell lines were used: MDA-MB231, breast adenocarcinoma (ATCC #HTB-26), SW780, bladder transitional cell carcinoma (kindly provided by Dr. Lars Dyrskjøt Andersen, Department of Molecular Medicine, Aarhus University Hospital, Skejby, Denmark) [41], H69, small cell lung carcinoma (kindly provided by the Department of Radiation Biology, Copenhagen University Hospital, Denmark) [42], and HT29, colorectal adenocarcinoma (ATCC #HTB-38). HDF-n, a primary normal human dermal fibroblast cell line (kindly provided by Dr. Marie-Pierre Rols, Institute of Pharmacology and Structural Biology, IPBS, Toulouse, France) [35] was also used in this study. MDA-MB231, SW780, and HDF-n were grown in DMEM, and H69 and HT29 were grown in RPMI-1640 culture medium (Gibco, Invitrogen) with 10% fetal calf serum (Gibco, Invitrogen), 100 U/ml penicillin, and 100 μg/ml streptomycin and kept at 37°C and 5% CO_2_.

Cells were tested negative for mycoplasma using MycoAlert mycoplasma detection kit (Lonza). Cancer cell lines were tested negative for infection using rapid MAP27 panel (Taconic), and authenticated by short tandem repeat (STR) profiling (LGC Standards) showing perfect match for SW780, H69, and HT29. The MDA-MB231 cell line matched in 7 of 9 profile loci (loci D7 and VWA have lost a peak) assessed not to influence the results of this study.

### Tumor studies in vivo

*In vivo* experiments were conducted in accordance with European Convention for the Protection of Vertebrate Animals used for Experimentation and with permission from the Danish Animal Experiments Inspectorate (# 2012-15-2934-00091).

Experiments were performed as described previously [26]. Briefly, 5.0 x 10^6^ cells in 100 μl PBS were injected subcutaneously in the flank of NMRI-*Foxn1*^*nu*^ mice (9–19 weeks old, males and females, 25–44 g (median 33 g) at treatment time, bred at Department of Oncology, Herlev Hospital, Denmark). Tumor volume was calculated as *axb*^*2*^*xπ/6*, where *a* is the longest diameter and *b* is the longest diameter perpendicular to *a*. At a tumor volume above 85 mm^3^, mice were randomized into the different groups according to a predefined randomization scheme. Thus, animals were randomized into the different treatment groups immediately before treatment securing similar weight and health status in all treatment groups.

Animals were housed in 425x266x150 mm cages with polyester filter sheet top (Scanbur) with environmental enrichment in groups of up to 8 mice with access to rat/mouse food (Altromin #1314, Brogaarden) and water *ad libitum*. The environmental conditions were a temperature of 22.3°C (SD 0.6°C), humidity of 48% (SD 7%), and a 12:12 dark:light cycle. Before treatment, mice were anesthetized (also untreated controls) by intraperitoneal injection of hypnorm and midazolam, and treatment was performed during the day in a laboratory in the animal facility. Tumors treated with electroporation were applied to 8 pulses of 1.0 kV/cm (applied voltage to electrode distance ratio), 100 μs, and 1 Hz using a 6 mm plate electrode and a square wave electroporator (Cliniporator, IGEA). Contact between electrode and tumor was ensured by ultrasound gel.

Humane endpoints were used to determine end of the experiments (i.e. tumors larger than 905 mm^3^, body weight decrease larger than 20% from pre-treatment, or sickness), where mice were euthanized by cervical dislocation. Mice were monitored daily for discomfort and sickness, three times a week for tumor size, and once a week for body weight.

### Effect on tumor volume of treatment combined with metformin

The SW780 cell line was used for this experiment since this cell line has been shown to be least sensitive to calcium electroporation of the four cancer cell lines when treated *in vivo* [43]. It has previously been shown that SW780 cells are sensitive to metformin [44]. Mice were randomized into 8 groups treated with (i) 168 mM CaCl_2_ injection with or without electroporation or (ii) physiological saline injection with or without electroporation; all treated either with or without metformin. Tumors were injected with CaCl_2_ or physiological saline volumes equivalent to half the tumor volume.

Before treatment (3–4 hours) mice were injected intraperitoneally (ip) with metformin (250 mg/kg) or PBS (10 ml/kg). Metformin groups were given metformin in the drinking water (2 mg/ml) for 2 weeks after treatment to sustain the expected effect of metformin on the ATP level in the tumors. The drug was active since we observed side effects on the mice as described in the Results and Discussion section. Before treatment and three times a week after treatment, tumor volume was measured using a Vernier caliper.

### Effect on intracellular ATP level of treatment combined with metformin

All four cancer cell lines were used for this experiment. Mice were injected with 5.0 x 10^6^ cells in 100 μl PBS as described above, except for the SW780 cells where 4.8 x 10^6^ cells in 100μl PBS were injected. Mice were randomized into the same 8 groups as described above, and injected ip 3–4 hours before treatment with metformin (250 mg/kg) or PBS (10 ml/kg). Around 1 hour after treatment with CaCl_2_ or NaCl with or without electroporation tumors were removed, halved, and put in 1 ml ice cold 5% TCA overnight. Tumors were lysed at 30 Hz for 4–10 min using a Tissue Lyser (Qiagen), centrifuged at 500 g for 2 min, and supernatant was diluted 7500 times in Tris-Acetate-EDTA (TAE). ATP content was determined using ELITEN ATP assay (Promega) and light emission was measured using LUMIstar (Ramcon).

### Viability in vitro

After harvesting, cells were washed and diluted to 6.1x10^6^ cell/ml in HEPES buffer (10mM HEPES (Lonza), 250 mM sucrose, and 1 mM MgCl_2_ in sterile water), and cooled on ice. The 4 mm aluminum electrode cuvettes (Molecular BioProducts, Inc.) placed in a cooling block, and 30 μl CaCl_2_ (final concentration of 0.25–5.0 mM) or HEPES buffer for controls were added to the cuvettes followed by addition of 270 μl cells. Cells incubated in the cuvettes for 5 min before electroporation or not. Cells were exposed to 8 pulses of 99 μs, 1 Hz, and 1.2 kV/cm (applied voltage to electrode distance ratio) using a square wave electroporator (BTX T820). After this, the cells incubated in the cuvettes at 37°C and 5% CO_2_ for 20 min, before they were transferred to culture medium and seeded in 96-well plates (3.1x10^4^ cells per 100 μl). MTS assay was performed 1 day after treatment using Multiskan-Ascent ELISA reader (Thermo Labsystems).

### ATP assay in vitro

Cells were treated as described above with 0, 1, 3, and 5 mM calcium with or without electroporation. Cells were seeded in white 96-well plates as described above and lysed 1, 2, 4, and 8 hours after treatment before ATP content was determined using ENLITEN ATP assay (Promega) and LUMIstar luminometer (Ramcon).

### Statistics

Statistical analyses were performed using SAS software (version 9.2).

Differences in cell viability *in vitro* and differences in intracellular ATP level *in vitro* and *in vivo* were assessed by 2-way analysis of variance (ANOVA) with post least-squares-means test with Bonferroni correction. ATP level in HT29 and MDA-MB231 tumors were log transformed before analysis.

Difference in survival after treatment was assessed using a Log-Rank test with death due to tumor size (above 905 mm^3^) defined as event and death due to disease was censored.

SPSS software (version 19) was used to evaluate difference in tumor volume after treatment with linear mixed model.

## Results and discussion

### Calcium electroporation combined with metformin treatment—Effect on tumor volume

**Fig 1:**
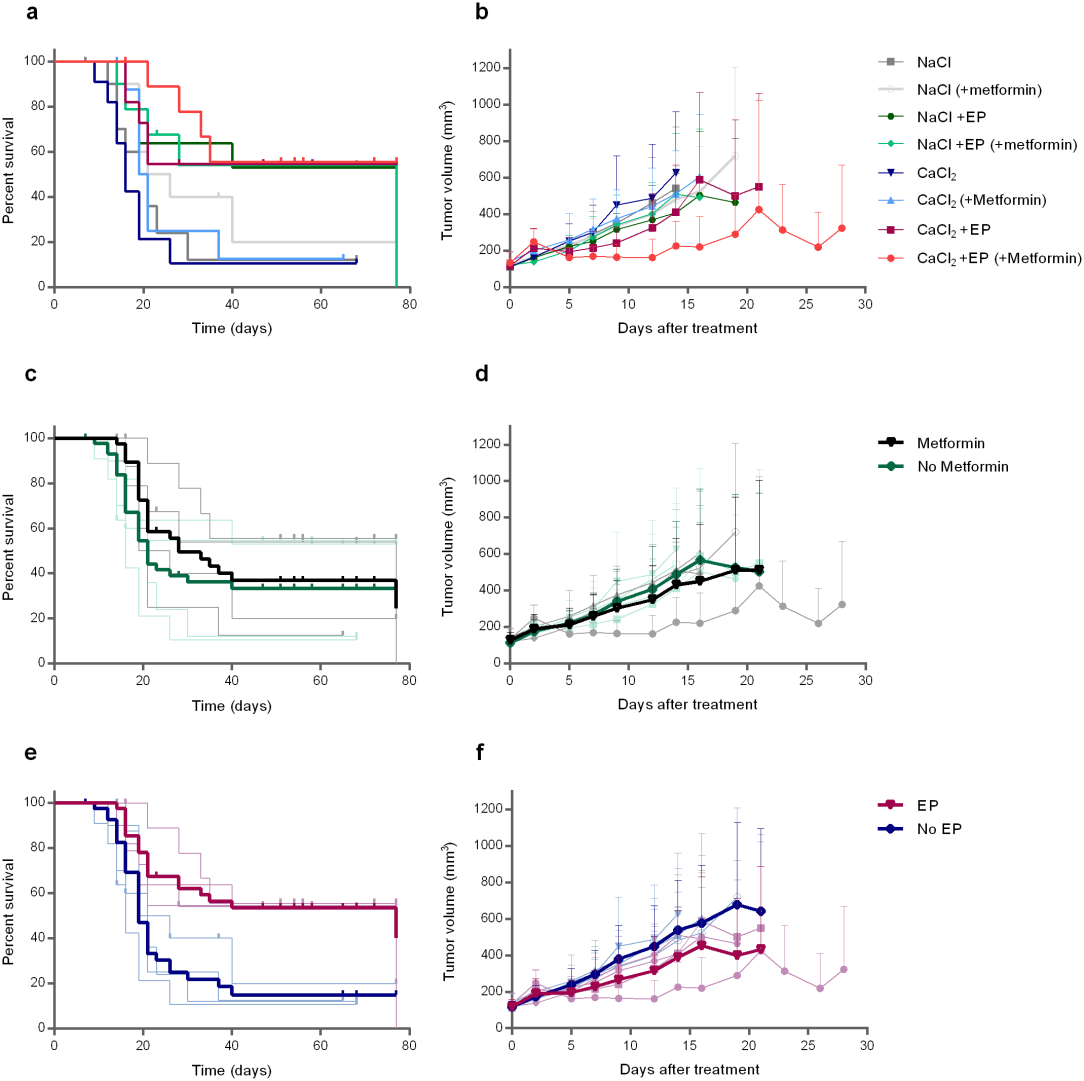
Effect of calcium electroporation and metformin treatment. SW780 (human bladder cancer) tumors treated with 168mM calcium or physiological saline with or without electroporation (EP) and with or without metformin treatment. Kaplan Meier curves after treatment shown for all treatment groups (a), and for pooled data for groups treated with (black) or without (green) metformin (c), as well as for groups treated with (red) or without (blue) electroporation (e). Death due to tumor size (above 905mm^3^) was defined as event and death due to disease was censored. Tumor volume in days after treatment shown for each treatment group (b), and for pooled data for groups treated with (black) or without (green) metformin (d), as well as for groups treated with (red) or without (blue) electroporation (f). The original treatment groups treated with (gray) or without (light green) metformin (c-d), as well as for groups treated with (light red) or without (light blue) electroporation (e-f) is shown in faded colors in the background. Mean + SD, n > 7 is shown in the graph. n = 9–11 before treatment.

To investigate if a systemic metformin treatment, which affects cell metabolism by inhibiting the mitochondrial ATP production [37], could increase the effect of the local treatment with calcium electroporation we tested this combination on SW780 (bladder cancer) tumors in nude mice (Fig 1b). The lowest tumor volume was seen in mice treated with calcium electroporation and metformin, however not significantly lower than in mice treated with calcium electroporation (p = 0.056; Fig 1b). Mice treated with calcium electroporation had significantly lower tumor volume than mice treated with calcium alone (p<0.05), similar to previous findings in a different tumor model [26]. This shows that calcium electroporation is effective in different tumor types. Looking at survival after treatment (Fig 1a), no significant difference in survival was seen between calcium electroporation with or without metformin treatment. For further analyses, the results for all mice were pooled into two groups treated with or without metformin (Fig 1c and 1d) showing no difference between the two groups in the effect after treatment on neither tumor volume nor survival. When pooling the data into two groups treated with or without electroporation, a significantly lower tumor volume (p<0.0001) and longer survival (p<0.001) was shown for mice in the electroporation group (Fig 1e and 1f), demonstrating that electroporation, as expected, increases the effect of the drugs.

The calcium concentration used *in vivo* was 168 mM, much higher than the concentrations causing cell death *in vitro* (>0.5–1.0 mM depending on the cell line used), due to the very small extracellular space in tumors compared with *in vitro*, as well as expected dilution to the remaining extracellular volume of the animal.

### Effect on intracellular ATP level after calcium electroporation combined with metformin treatment

**Fig 2:**
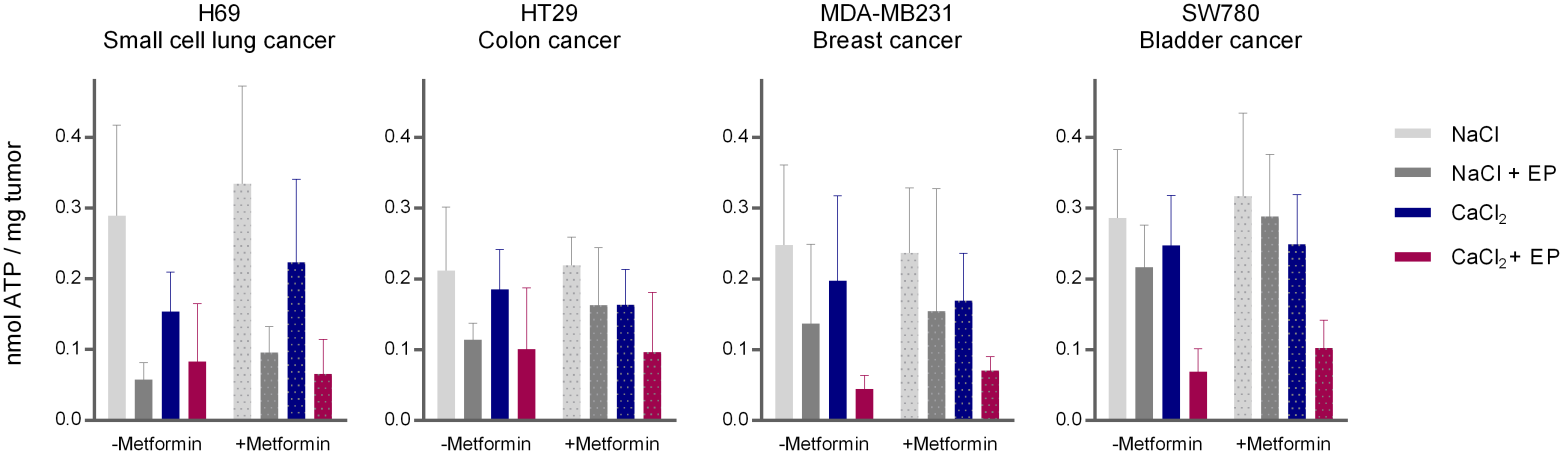
Intracellular ATP in tumors after treatment with calcium electroporation and metformin treatment. Four different human tumor types; H69, a small cell lung cancer, HT29, a colon cancer, MDA-MB231, a breast cancer, and SW780, a bladder cancer treated with 168mM calcium or physiological saline with or without electroporation (EP). Metformin or PBS was injected ip 3–4 hours before treatment, tumors were removed around 1 hour after treatment, and ATP level was measured after lysis overnight. Mean + SD, n = 5–7 (H69), n = 6–8 (HT29), n = 4–5 (MDA-MB231), n = 4–6 (SW780).

The intracellular ATP level in tumors was measured 1 hour after treatment to investigate if calcium electroporation *in vivo* induced ATP depletion like *in vitro*, and to test if the additional metformin treatment further decreased the ATP level (Fig 2). We measured the ATP level in four different tumors (small cell lung cancer, colon cancer, breast cancer, and bladder cancer) treated with calcium electroporation with or without additional metformin treatment.

The results showed that addition of metformin did not significantly decrease the ATP level. Since neither tumor size, survival, nor ATP level was significantly affected by addition of metformin injection this might be due to the chosen concentrations used, and it could be hypothesized that changing the concentration of metformin could have an effect. An effect might have been shown by increasing the concentration but 20 out of 89 mice treated with metformin had a slightly lowered body temperature 3 hours after injection, and were put in a cage on a warming block. This indicate that the used concentration was toxic and too high, thus, we did not try with a higher concentration of metformin due to the observed adverse effects.

**Fig 3:**
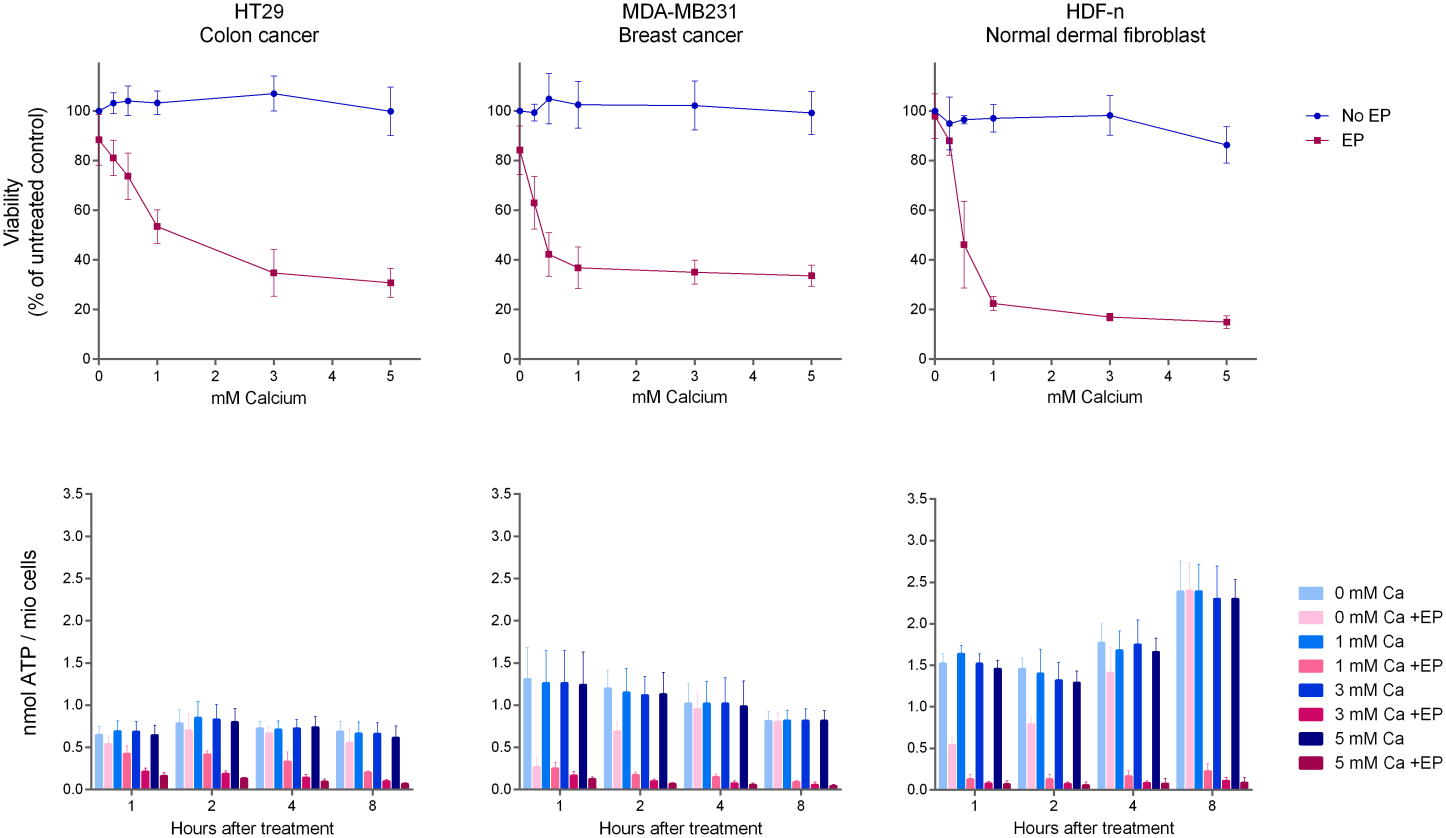
Viability and intracellular ATP level after calcium electroporation in vitro. Two human cancer cell lines (HT29, colon cancer and MDA-MB231, breast cancer) and a normal primary human dermal fibroblast cell line (HDF-n) were treated with increasing concentration of calcium with or without electroporation (EP). Viability was measured 1 day after treatment and intracellular ATP level was measured 1, 2, 4, and 8 hours after treatment. Mean +/- SD, n = 4–9.

In control tumors treated only with physiological saline the ATP level was between 0.21–0.29 nmol/mg tumor. When treating tumors with calcium electroporation, the ATP level was significantly lower than in controls (p<0.05 in all tumor types), as seen *in vitro* (Fig 3 and S1 Fig). Treatment with physiological saline and electroporation significantly reduced intracellular ATP level only in the small cell lung cancer tumors (p<0.01). *In vitro*, it was also shown that electroporation alone significantly decreased the ATP level 1 hour after treatment in H69 (small cell lung cancer), MDA-MB231 (breast cancer) cells, and HDF-n (normal fibroblasts) (p<0.0001). This decrease after electroporation alone is likely due to direct loss of ATP through the permeabilized membrane and re-establishment of ionic homeostasis using ATP dependent pumps. In some of the cell lines and tumors, no significant decrease in ATP level after electroporation alone was observed, which could be due to no or lower direct loss of ATP, or due to re-establishment of the ATP level within 1 hour after treatment.

It has previously been shown that electroporation cause a vascular effect in tumors, where the blood flow in the treated area is reduced around 60% for up to 2 hours after treatment [45, 46]. This reduced blood flow likely causes limited access of nutrients needed for the cells to produce ATP, thereby increasing the effect of calcium electroporation. This effect might have been seen if the tumors were removed at a later time after treatment.

### Viability in vitro after calcium electroporation

Viability in different cancer cell lines and a primary normal cell line after treatment with increasing calcium concentration with or without electroporation is depicted in Fig 3 and in S1 Fig. As previously shown [26, 36], treatment with calcium alone did not affect viability whereas calcium electroporation induced dramatic decrease in viability (p<0.0001 for concentrations above 0.5 mM for H69, HDF-n, and MDA-MB231; p<0.0001 for concentrations above 1 mM for HT29 and SW780) with concentrations causing 50% cell death ranging from 0.37–1.25 mM CaCl_2_ in the five cell lines (Table 1). Viability of normal primary fibroblasts decreased (50% cell death at 0.48 mM) after calcium electroporation, like viability of the cancer cells. However, it has been shown that normal primary fibroblasts were much less affected than cancer cell lines after calcium electroporation, when treated as spheroids (a multicellular 3D *in vitro* model) [35]. This might be due to the model systems used, where cells in a spheroid are densely packed and forming intercellular junction and anatomical communication [47], unlike cells in suspension that are separate from each other, are not oriented, and have a very large extracellular volume. Cells in spheroids better mimic cells in tissue and it has recently been shown that normal tissue is less affected that tumor tissue to calcium electroporation [43].

**Table 1:** Sensitivity to calcium electroporation *in vitro* measured by 50% cell death.

Calcium concentration causing 50% cell death	
Cell line	mM
MDA-MB231	0.37
HDF-n	0.48
H69	0.56
SW780	0.80
HT29	1.25

Calcium concentration, where 50% of cells were dead, was estimated from graphs in Fig 3 and S1 Fig. Viability of untreated control was denoted as 100%.

Electroporation alone also affected the cells where viability decreased the most in H69 cells (27% decrease in viability after electroporation, p<0.0001), followed by SW780 (19%, p = 0.0797), MDA-MB231 (16%, p = 0.1191), HT29 (12%, p = 1), and the normal cell line HDF-n, where viability only decreased 2% after electroporation (p = 1).

### Intracellular ATP level in vitro after calcium electroporation

To investigate if viability of cells after calcium electroporation is related to the intracellular calcium concentration before or after treatment, the intracellular ATP level was determined 1, 2, 4, and 8 hours after treatment with increasing CaCl_2_ concentration (0–5 mM) with or without electroporation. The ATP level decreased significantly after calcium electroporation compared with treatment without electroporation (p<0.0001; Fig 3), as previously shown (S1 Fig) [26, 36]. It has also previously been shown that the ATP level decreases after electroporation alone [26, 36], which is also shown here, where the ATP level decreased 1 hour after treatment with electroporation alone in HDF-n, and MDA-MB231 cells (p<0.0001). This is likely due to direct loss of ATP through the permeabilized cell membrane as well as ATP used to re-establish ion homeostasis after permeabilization by employing ATP dependent membrane pumps; however, the cells re-established the ATP level over time where no significant difference was seen 8 hours after treatment.

Increasing calcium concentrations were used in this experiment, and the ATP level was significantly lower when electroporating with 5 mM calcium than when electroporating with 1 mM calcium in all cell lines 2, 4, and 8 hours after treatment (p<0.05), except in the H69 cell line (p = 1.000) as previously shown (S1 Fig), indicating a dose response on the ATP depletion caused by calcium electroporation. The ATP level was very low in the H69 cell line after treatment with 1 mM calcium electroporation, and this is probably the reason why no further decrease in ATP level was seen in this cell line.

The intracellular ATP depletion after calcium electroporation in relation to the viability of the cells after treatment was investigated. In untreated cells, the intracellular ATP level varied between the cell lines; H69 (previously published) had the lowest intracellular ATP concentration of 0.26 nmol ATP per million cells followed by HT29 (0.65 nmol ATP/mio cells), MDA-MB231 (1.31 nmol ATP/mio cells), SW780 (1.50 nmol ATP/mio cells; previously published), and HDF-n with the highest intracellular ATP concentration of the tested cell lines (1.52 nmol ATP/mio cells). The intracellular ATP level in cells before treatment might affect their ability to survive treatment with calcium electroporation, since the treatment is associated with ATP depletion. However, there is no correlation between intracellular ATP level in untreated cells and sensitivity to treatment *in vitro*, measured in calcium concentrations causing 50% cell death in combination with electroporation (Table 1). An important point might be that not only the ATP depletion affects viability, but also the cells sensitivity to this depletion.

## Conclusion

This study showed that systemic metformin treatment did not increase the effect of calcium electroporation *in vivo*, neither on tumor size, survival, nor ATP level in the model tested. We also showed that calcium electroporation was associated with significant ATP depletion both *in vitro* and *in vivo*, and consistently in four different malignant cell lines. However, we did not see a relation between ATP level in cells before treatment and sensitivity to the treatment. We have previously shown that calcium electroporation induces tumor necrosis, and was presented as a possible novel anti-cancer treatment. In this study, we have not shown increased effect when combining the treatment with metformin but further research to understand the mechanism behind calcium electroporation could lead to optimization of this promising novel anti-cancer treatment.

## Supporting information

S1 Fig Viability and intracellular ATP level after calcium electroporation in vitro. Two human cancer cell lines (H69, small cell lung cancer and SW780, bladder cancer) were treated with increasing concentration of calcium with or without electroporation (EP). Viability was measured 1 day after treatment and intracellular ATP level was measured 1, 2, 4, and 8 hours after treatment. Mean +/- SD, n = 4–9. The ATP data has previously been published but illustrated differently [36]. The data is also presented in this study to compare ATP level in five cell lines (Fig 3) as well as to compare the ATP levels with viability in each cell line.
(TIF)

## References

[1] Rols MP, Teissie J. Electropermeabilization of mammalian cells. Quantitative analysis of the phenomenon. Biophys J. 1990;58(5):1089–1098. doi: 10.1016/S0006-3495(90)82451-6 2291935 10.1016/S0006-3495(90)82451-6 PMC1281055

[2] Li S. Electroporation Protocols—Preclinical and clinical gene medicine. 1st ed Totowa, New Jersey: Humana Press; 2008.

[3] Kee ST, Gehl J, Lee EW. Clinical Aspects of Electroporation. New York: Springer; 2011.

[4] Gehl J, Skovsgaard T, Mir LM. Enhancement of cytotoxicity by electropermeabilization: an improved method for screening drugs. Anti-Cancer Drugs. 1998;9(4):319–325. 9635922 10.1097/00001813-199804000-00005

[5] Jaroszeski MJ, Dang V, Pottinger C, Hickey J, Gilbert R, Heller R. Toxicity of anticancer agents mediated by electroporation in vitro. Anti-Cancer Drugs. 2000;11(3):201–208. 10831279 10.1097/00001813-200003000-00008

[6] Orlowski S, Belehradek J, Paoletti C, Mir LM. Transient Electropermeabilization of Cells in Culture—Increase of the Cyto-Toxicity of Anticancer Drugs. Biochemical Pharmacology. 1988;37(24):4727–4733. 2462423 10.1016/0006-2952(88)90344-9

[7] Sersa G, Cemazar M, Miklavcic D. Antitumor effectiveness of electrochemotherapy with cis-diamminedichloroplatinum(II) in mice. Cancer Res. 1995;55(15):3450–3455. 7614485

[8] Vasquez JL, Gehl J, Hermann GG. Electroporation enhances mitomycin C cytotoxicity on T24 bladder cancer cell line: a potential improvement of intravesical chemotherapy in bladder cancer. Bioelectrochemistry. 2012;88:127–133. doi: 10.1016/j.bioelechem.2012.08.001 22940093 10.1016/j.bioelechem.2012.08.001

[9] Vasquez JL, Ibsen P, Lindberg H, Gehl J. In vitro and in vivo experiments on electrochemotherapy for bladder cancer. JUrol. 2015;193(3):1009–1015. 25245485 10.1016/j.juro.2014.09.039

[10] Belehradek M, Domenge C, Luboinski B, Orlowski S, Belehradek J Jr., Mir LM. Electrochemotherapy, a new antitumor treatment. First clinical phase I-II trial. Cancer. 1993;72(12):3694–3700. 7504576 10.1002/1097-0142(19931215)72:12<3694::aid-cncr2820721222>3.0.co;2-2

[11] Campana LG, Testori A, Mozzillo N, Rossi CR. Treatment of metastatic melanoma with electrochemotherapy. J Surg Oncol. 2014;109(4):301–307. 24678530 10.1002/jso.23512

[12] Curatolo P, Miraglia E, Rotunno R, Calvieri S, Giustini S. Electrochemotherapy: a valid treatment for Gorlin-Goltz syndrome. Acta Dermatovenerol Croat. 2013;21(2):132–133. 24001423

[13] Curatolo P, Quaglino P, Marenco F, Mancini M, Nardo T, Mortera C, et al Electrochemotherapy in the treatment of Kaposi sarcoma cutaneous lesions: a two-center prospective phase II trial. AnnSurgOncol. 2012;19(1):192–198. 10.1245/s10434-011-1860-7 21822561

[14] Heller R, Jaroszeski MJ, Reintgen DS, Puleo CA, DeConti RC, Gilbert RA, et al Treatment of cutaneous and subcutaneous tumors with electrochemotherapy using intralesional bleomycin. Cancer. 1998;83(1):148–157. 9655305 10.1002/(sici)1097-0142(19980701)83:1<148::aid-cncr20>3.0.co;2-w

[15] Matthiessen LW, Chalmers RL, Sainsbury DC, Veeramani S, Kessell G, Humphreys AC, et al Management of cutaneous metastases using electrochemotherapy. Acta Oncol. 2011;50(5):621–629. doi: 10.3109/0284186X.2011.573626 21574833 10.3109/0284186X.2011.573626 PMC3130997

[16] Matthiessen LW, Johannesen HH, Hendel HW, Moss T, Kamby C, Gehl J. Electrochemotherapy for large cutaneous recurrence of breast cancer: a phase II clinical trial. Acta Oncol. 2012;51(6):713–721. doi: 10.3109/0284186X.2012.685524 22731832 10.3109/0284186X.2012.685524

[17] Fini M, Salamanna F, Parrilli A, Martini L, Cadossi M, Maglio M, et al Electrochemotherapy is effective in the treatment of rat bone metastases. Clin Exp Metastasis. 2013;30(8):1033–1045. doi: 10.1007/s10585-013-9601-x 23832763 10.1007/s10585-013-9601-x

[18] Granata V, Fusco R, Piccirillo M, Palaia R, Petrillo A, Lastoria S, et al Electrochemotherapy in locally advanced pancreatic cancer: Preliminary results. Int J Surg. 2015;18:230–236. doi: 10.1016/j.ijsu.2015.04.055 25917204 10.1016/j.ijsu.2015.04.055

[19] Forde PF, Sadadcharam M, Bourke MG, Conway TA, Guerin SR, de Kruijf M, et al Preclinical evaluation of an endoscopic electroporation system. Endoscopy. 2016;48(5):477–483. doi: 10.1055/s-0042-101343 27042930 10.1055/s-0042-101343

[20] Landstrom FJ, Reizenstein JA, Nilsson CO, Beckerath MV, Lofgren AL, Adamsson GB, et al Electrochemotherapy—possible benefits and limitations to its use in the head and neck region. Acta Otolaryngol. 2015;135(1):90–95. doi: 10.3109/00016489.2014.947655 25496181 10.3109/00016489.2014.947655

[21] Davalos RV, Bhonsle S, Neal RE 2nd. Implications and considerations of thermal effects when applying irreversible electroporation tissue ablation therapy. Prostate. 2015;75(10):1114–1118. doi: 10.1002/pros.22986 25809014 10.1002/pros.22986 PMC6680146

[22] Neal RE 2nd, Garcia PA, Kavnoudias H, Rosenfeldt F, McLean CA, Earl V, et al In vivo irreversible electroporation kidney ablation: experimentally correlated numerical models. IEEE Trans Biomed Eng. 2015;62(2):561–569. doi: 10.1109/TBME.2014.2360374 25265626 10.1109/TBME.2014.2360374

[23] Neal RE 2nd, Millar JL, Kavnoudias H, Royce P, Rosenfeldt F, Pham A, et al In vivo characterization and numerical simulation of prostate properties for non-thermal irreversible electroporation ablation. Prostate. 2014;74(5):458–468. doi: 10.1002/pros.22760 24442790 10.1002/pros.22760

[24] Beebe SJ, Fox PM, Rec LJ, Willis EL, Schoenbach KH. Nanosecond, high-intensity pulsed electric fields induce apoptosis in human cells. FASEB J. 2003;17(11):1493–1495. doi: 10.1096/fj.02-0859fje 12824299 10.1096/fj.02-0859fje

[25] Pakhomova ON, Gregory B, Semenov I, Pakhomov AG. Calcium-mediated pore expansion and cell death following nanoelectroporation. Biochim Biophys Acta. 2014;1838(10):2547–2554. doi: 10.1016/j.bbamem.2014.06.015 24978108 10.1016/j.bbamem.2014.06.015 PMC4125538

[26] Frandsen SK, Gissel H, Hojman P, Tramm T, Eriksen J, Gehl J. Direct therapeutic applications of calcium electroporation to effectively induce tumor necrosis. Cancer Res. 2012;72(6):1336–1341. doi: 10.1158/0008-5472.CAN-11-3782 22282658 10.1158/0008-5472.CAN-11-3782

[27] Berridge MJ, Bootman MD, Roderick HL. Calcium signalling: Dynamics, homeostasis and remodelling. Nature Reviews Molecular Cell Biology. 2003;4(7):517–529. doi: 10.1038/nrm1155 12838335 10.1038/nrm1155

[28] Brini M, Carafoli E. Calcium signalling: a historical account, recent developments and future perspectives. Cell MolLife Sci. 2000;57(3):354–370. 10.1007/PL00000698 PMC11146899 10823237

[29] Clapham DE. Calcium signaling. Cell. 2007;131(6):1047–1058. doi: 10.1016/j.cell.2007.11.028 18083096 10.1016/j.cell.2007.11.028

[30] Zhivotovsky B, Orrenius S. Calcium and cell death mechanisms: A perspective from the cell death community. Cell Calcium. 2011;50(3):211–221. doi: 10.1016/j.ceca.2011.03.003 21459443 10.1016/j.ceca.2011.03.003

[31] Aung CS, Ye W, Plowman G, Peters AA, Monteith GR, Roberts-Thomson SJ. Plasma membrane calcium ATPase 4 and the remodeling of calcium homeostasis in human colon cancer cells. Carcinogenesis. 2009;30(11):1962–1969. doi: 10.1093/carcin/bgp223 19755660 10.1093/carcin/bgp223

[32] Papp B, Brouland JP, Arbabian A, Gelebart P, Kovacs T, Bobe R, et al Endoplasmic reticulum calcium pumps and cancer cell differentiation. Biomolecules. 2012;2(1):165–186. doi: 10.3390/biom2010165 24970132 10.3390/biom2010165 PMC4030869

[33] Frandsen SK, Gissel H, Hojman P, Eriksen J, Gehl J. Calcium electroporation in three cell lines: a comparison of bleomycin and calcium, calcium compounds, and pulsing conditions. BiochimBiophysActa. 2014;1840(3):1204–1208. 10.1016/j.bbagen.2013.12.003 24342489

[34] Zielichowska A, Daczewska M, Saczko J, Michel O, Kulbacka J. Applications of calcium electroporation to effective apoptosis induction in fibrosarcoma cells and stimulation of normal muscle cells. Bioelectrochemistry. 2016;109:70–78. doi: 10.1016/j.bioelechem.2016.01.005 26874618 10.1016/j.bioelechem.2016.01.005

[35] Frandsen SK, Gibot L, Madi M, Gehl J, Rols MP. Calcium Electroporation: Evidence for Differential Effects in Normal and Malignant Cell Lines, Evaluated in a 3D Spheroid Model. PLoS One. 2015;10(12):e0144028 doi: 10.1371/journal.pone.0144028 26633834 10.1371/journal.pone.0144028 PMC4669124

[36] Hansen EL, Sozer EB, Romeo S, Frandsen SK, Vernier PT, Gehl J. Dose-dependent ATP depletion and cancer cell death following calcium electroporation, relative effect of calcium concentration and electric field strength. PLoS One. 2015;10(4):e0122973 doi: 10.1371/journal.pone.0122973 25853661 10.1371/journal.pone.0122973 PMC4390219

[37] Pryor R, Cabreiro F. Repurposing metformin: an old drug with new tricks in its binding pockets. Biochem J. 2015;471(3):307–322. doi: 10.1042/BJ20150497 26475449 10.1042/BJ20150497 PMC4613459

[38] Cho SW, Yi KH, Han SK, Sun HJ, Kim YA, Oh BC, et al Therapeutic potential of metformin in papillary thyroid cancer in vitro and in vivo. Mol Cell Endocrinol. 2014;393(1–2):24–29. doi: 10.1016/j.mce.2014.05.021 24905037 10.1016/j.mce.2014.05.021

[39] El-Mir MY, Nogueira V, Fontaine E, Averet N, Rigoulet M, Leverve X. Dimethylbiguanide inhibits cell respiration via an indirect effect targeted on the respiratory chain complex I. J Biol Chem. 2000;275(1):223–228. 10617608 10.1074/jbc.275.1.223

[40] Owen MR, Doran E, Halestrap AP. Evidence that metformin exerts its anti-diabetic effects through inhibition of complex 1 of the mitochondrial respiratory chain. Biochem J. 2000;348 Pt 3:607–614. 10839993 PMC1221104

[41] Herbsleb M, Birkenkamp-Demtroder K, Thykjaer T, Wiuf C, Hein AM, Orntoft TF, et al Increased cell motility and invasion upon knockdown of lipolysis stimulated lipoprotein receptor (LSR) in SW780 bladder cancer cells. BMCMedGenomics. 2008;1:31. 10.1186/1755-8794-1-31 PMC2492871 18647386

[42] Gjetting T, Arildsen NS, Christensen CL, Poulsen TT, Roth JA, Handlos VN, et al In vitro and in vivo effects of polyethylene glycol (PEG)-modified lipid in DOTAP/cholesterol-mediated gene transfection. IntJNanomedicine. 2010;5:371–383. 10.2147/ijn.s10462 PMC2950395 20957159

[43] Frandsen SK, Krüger MB, Mangalanathan UM, Tramm T, Mahmood F, Novak I, et al Normal and malignant cells exhibit differential responses to calcium electroporation Cancer Res. 2017;Accepted for publication. 10.1158/0008-5472.CAN-16-1611 28760856

[44] Guo LS, Li HX, Li CY, Zhang SY, Chen J, Wang QL, et al Vitamin D3 enhances antitumor activity of metformin in human bladder carcinoma SW-780 cells. Pharmazie. 2015;70(2):123–128. 25997253

[45] Jarm T, Cemazar M, Miklavcic D, Sersa G. Antivascular effects of electrochemotherapy: implications in treatment of bleeding metastases. Expert Rev Anticancer Ther. 2010;10(5):729–746. doi: 10.1586/era.10.43 20470005 10.1586/era.10.43

[46] Markelc B, Sersa G, Cemazar M. Differential mechanisms associated with vascular disrupting action of electrochemotherapy: intravital microscopy on the level of single normal and tumor blood vessels. PLoS One. 2013;8(3):e59557 doi: 10.1371/journal.pone.0059557 23555705 10.1371/journal.pone.0059557 PMC3608732

[47] Sutherland RM. Cell and environment interactions in tumor microregions: the multicell spheroid model. Science. 1988;240(4849):177–184. 2451290 10.1126/science.2451290

